# Association of Gut Microbiota and Metabolites With Disease Progression in Children With Biliary Atresia

**DOI:** 10.3389/fimmu.2021.698900

**Published:** 2021-09-23

**Authors:** Wei Song, Li-Ying Sun, Zhi-Jun Zhu, Lin Wei, Wei Qu, Zhi-Gui Zeng, Ying Liu, Hai-Ming Zhang, Wei Guo

**Affiliations:** ^1^ Liver Transplantation Center, National Clinical Research Center for Digestive Diseases, Beijing Friendship Hospital, Capital Medical University, Beijing, China; ^2^ Clinical Center for Pediatric Liver Transplantation, Capital Medical University, Beijing, China; ^3^ Department of Intensive Care Unit, Beijing Friendship Hospital, Capital Medical University, Beijing, China; ^4^ Hangkong Hospital, China Capital University, Beijing, China

**Keywords:** gut microbiota, metagenomic sequencing, metabolites, ultra-performance liquid chromatography/tandem mass spectrometry, biliary atresia (BA)

## Abstract

**Background and Aims:**

Biliary atresia is the most common cause of liver disease and liver transplantation in children. The accumulation of bile acids in hepatocytes and the stimulation of the intestinal microbiome can aggravate the disease progression. This study investigated changes in the composition of the gut microbiota and its metabolites in biliary atresia and the possible effects of these changes on disease progression.

**Methods:**

Stool samples of biliary atresia at different disease stages and matched control individuals were collected (early stage: 16 patients, 16 controls; later stage: 16 patients, 10 controls). Metagenomic sequencing was performed to evaluate the gut microbiota structure. Untargeted metabolomics was performed to detect and analyze the metabolites and bile acid composition.

**Results:**

A disturbed gut microbiota structure occurred in the early and later stages of biliary atresia. *Klebsiella*, *Streptococcus*, *Veillonella*, and *Enterococcus* have always been dominant. The abundance of *V. atypica* displayed significant changes between the early and later stages of biliary atresia. Combined with clinical indicators, Spearman’s analysis showed that *Klebsiella* and *Veillonella atypica* strongly correlated with liver enzymes. *Enterococcus faecium* had an enormously positive relationship with lithocholic acid derivatives. Metabolites involved in tryptophan metabolism were changed in the patients with biliary atresia, which had a significant association with stool *V. atypica* and blood total bilirubin (*p* < 0.05).

**Conclusions:**

The liver damage of biliary atresia was directly or indirectly exacerbated by the interaction of enriched *Klebsiella* (*K. pneumoniae*), *Veillonella* (*V. atypica*), and *Enterococcus* (*E. faecium*) with dysmetabolism of tryptophan and bile acid.

## Introduction

Biliary atresia (BA) is an idiopathic neonatal biliary disease characterized by progressive intrahepatic or extrahepatic bile duct inflammatory occlusion (1–3). Biliary atresia is the most common among the many conditions that cause neonatal cholestasis (25%–55%) (4–6). The reported incidence of biliary atresia is only 1 in 8,000 to 18,000 children but varies according to geography and ethnicity (5, 7). Prolonged neonatal jaundice, pale stools, and conjugated hyperbilirubinemia are typical biliary atresia symptoms (8). The disease can damage hepatocytes and lead to cirrhosis. There is no medical treatment for biliary atresia, with >50% of patients needing liver transplantation by 2 years of age (7, 9–11). Experimental and clinical studies have shown that viral infection triggers the destruction of the biliary epithelium and release of antigens, which trigger the Th1 immune response, release proinflammatory cytokines, and further damage the bile ducts. Infection by cytomegalovirus (CMV), rhinovirus, human herpes virus, human papillomavirus, adenovirus, Epstein–Barr virus (EBV), hepatitis B virus, parvovirus B19, and rotavirus in the liver and hepatobiliary tree may be associated with the occurrence of biliary atresia and other infantile obstructive cholangiopathies (12). Besides, previous research demonstrated that cholestasis, the accumulation of bile acids in the liver, fails to promote liver injury in the absence of the microbiome *in vivo* (13). Luo et al. (14) detected bacterial DNA from the gut in the blood of patients with biliary atresia, including *Escherichia coli*, *Klebsiella pneumoniae*, *Shigella flexneri*, and Enterobacteriaceae. These bacteria adhere to the bile duct epithelium *via* the surface protein adhesin and then damage the bile duct epithelium. Ahmed et al. (15) found that CD14 expression was more extensive in the liver tissues of children with biliary atresia than normal and disease controls. They considered that exposure to portal-derived LPS might lead to CD14 overexpression in biliary atresia. LPS, also named endotoxin, binds to its receptor CD14 and causes activation of macrophages with consequent release of cytokines such as interleukin (IL)-1, IL-6, tumor necrosis factor (TNF)-α, and interferon (IFN)-γ (16). Chou et al. (16) also found that the plasma levels of endotoxin and CD14 were higher in patients with biliary atresia. Based on previous literature and experiments, we hypothesized that the intestinal microbiome constitutes a causal factor in the bile duct and liver injury of biliary atresia.

The gut–liver axis is the anatomical and physiological bridge connecting the intestine and liver (17, 18). Gut microbes and their metabolites enter the blood circulation and bile to damage the biliary tract and hepatocytes through the gut–liver axis (19). Gut microbiota disorders have been found in various liver diseases such as alcoholic hepatitis, liver cirrhosis, hepatocellular carcinoma, ischemic liver injury, and liver graft rejection (20–23), which all show a particular influence on the occurrence or progression of the disease. Concerning biliary atresia, several studies have found that the intestinal flora is maladjusted, increasing the abundance of opportunistic pathogens (e.g., Proteobacteria*, Klebsiella*, *Enterococcus*, and *Streptococcus*) and decreasing that of butyrate-producing bacteria (24–26). However, the characteristics of intestinal metabolites in biliary atresia and changes in gut microbes during disease progression have not been studied.

In the current study, we characterized the structure of the gut microbes in the early and late stages of biliary atresia and searched for microbes related to disease progression. In addition, we quantitatively analyzed the metabolites in stool samples and examined the possible effects of these altered products on the disease.

## Materials and Methods

### Study Design and Sample Collection

We enrolled 16 patients with early-stage biliary atresia (age < 3 months) and 16 age-matched healthy control individuals. The diagnosis of biliary atresia was confirmed by intraoperative cholangiography and liver biopsy. The enrolled individuals met the following criteria: (1) no diarrhea or constipation within 4 weeks of the study and (2) no antibiotics and probiotics within 4 weeks. We enrolled 16 patients with late-stage biliary atresia (age <3 years) listed for liver transplantation at Beijing Friendship Hospital, Capital Medical University. The diagnosis and enrollment criteria were consistent with the early cohort. We also recruited 10 healthy control individuals. **Table S1** shows the detailed demographic information and hepatic function indices of the biliary atresia patients and healthy control individuals.

### Ethical Approval

The study was approved by the Ethical Committee of Beijing Friendship Hospital, Capital Medical University (Approval ID: 2019-P2–131-02), and informed consent was obtained from each participant’s guardians. Patient consent for publication was obtained.

### Sample Collection and DNA Extraction

Fecal samples from patients and healthy controls were all freshly collected and frozen at −80°C within four h after sampling. Bacterial DNA was extracted using the QIAamp Fast DNA Stool Mini Kit (51604; Qiagen, Hilden, Germany). One milliliter of Inhibitex Buffer and glass beads (0.5 mm diameter; Qiagen) were added to each 180–200 mg of feces. The mixture was homogenized twice at 60 Hz for 1 min with a FastPrep-24 (Aosheng Biotech, China). Subsequent steps of the DNA extraction protocol followed the manufacturer’s instructions for bacterial DNA extraction. The DNA concentration was measured with a NanoDrop (Thermo Scientific, Massachusetts, USA) and Qubit^®^ 2.0 (Invitrogen, Carlsbad, CA, USA), and the molecular size was estimated by agarose gel electrophoresis.

### Library Construction and Metagenomic Sequencing

Following the Illumina TruSeq DNA Sample Prep v2 Guide (San Diego, CA, USA), we constructed the DNA libraries with an approximately 500-bp insert size for each sample. All libraries’ quality was evaluated using an Agilent 2100 Bioanalyzer (Agilent Technologies, Wokingham, UK) and the Agilent 2100 DNA 1000 kit (27). The gut microbiome composition and function in feces were evaluated by metagenomic sequencing. All samples were subject to 150-bp paired-end sequencing on a HiSeq X Ten platform (Illumina).

### Bioinformatic Analysis of Metagenomic Sequencing

Illumina raw reads were screened according to the following criteria: (1) adaptor contamination reads were removed; (2) reads containing more than three ambiguous N bases were removed; (3) reads containing low-quality (Q < 20) bases were trimmed; and (4) reads containing less than 60% of high-quality bases (Phred score ≥20) were deleted.

Clean reads were subjected to bacterial genomes from the National Center for Biotechnology Information GenBank with SOAPaligner (version 2.21), and reads mapped to the host genome were abandoned.

For species classification, the NCBI database (National Center for Biological Information, http://www.ncbi.nlm.nih.gov) aligned the clean reads with known bacteria, fungi, viruses, and archaea by SOAPaligner 2.21. Concerning the functional profiles, the non-redundant genes were annotated against the KEGG (Kyoto Encyclopedia of Genes and Genomes) database (KEGG, http://www.genome.jp/kegg/) using BLAST (v. 2.2.28+). When the assembled protein sequence was similar (score ≥60 and E value < 1e–5) to a protein sequence in the database, the produced protein was considered to play the same role as the database protein. The relative abundance of all orthologous genes was accumulated to generate the relative lot of each KO (KEGG ortholog).

### Analysis of Untargeted Metabolomics

#### Metabolite Extraction

A 50-mg sample was accurately weighed, and the metabolites were extracted using a 400-µl methanol: water (4:1, v/v) solution. The mixture was allowed to settle at -20°C and treated by high-throughput tissue crusher Wonbio-96c (Shanghai Wanbo Biotechnology Co., Ltd.) at 50 Hz for 6 min, followed by vortex for 30 s and ultrasound at 40 kHz for 30 min at 5°C. The samples were placed at -20°C for 30 min to precipitate proteins. After centrifugation at 13,000g at 4°C for 15 min, the supernatant was carefully transferred to sample vials for LC-MS/MS analysis.

#### Quality Control Sample

As part of the system conditioning and quality control process, a pooled quality control sample (QC) was prepared by mixing equal volumes of all models. The QC samples were disposed of and tested in the same manner as the analytic samples. It helped represent the whole sample set, which would be injected at regular intervals (every eight samples) to monitor the stability of the analysis.

#### LC/MC Analysis

Chromatographic separation of the metabolites was performed on an ExionLC™ AD system (AB Sciex, USA) equipped with an ACQUITY UPLC BEH C18 column (100 mm × 2.1 mm i.d., 1.7 µm; Waters, Milford, USA). The mobile phases consisted of 0.1% formic acid in water with formic acid (0.1%) (solvent A) and 0.1% formic acid in acetonitrile:isopropanol (1:1, v/v) (solvent B). The solvent gradient changed according to the following conditions: from 0 to 3 min, 95% (A): 5% (B) to 80% (A): 20% (B); from 3 to 9 min, 80% (A): 20% (B) to 5% (A): 95% (B); from 9 to 13 min, 5% (A): 95% (B) to 5% (A): 95% (B); from 13 to 13.1 min, 5% (A): 95% (B) to 95% (A): 5% (B), from 13.1 to 16 min, 95% (A): 5% (B) to 95% (A): 5% (B) for equilibrating the systems. The sample injection volume was 20 μl, and the flow rate was set to 0.4 ml/min. The column temperature was maintained at 40°C. During the period of analysis, all these samples were stored at 4°C.

The UPLC system was coupled to a quadrupole time-of-flight mass spectrometer (Triple TOF™ 5600+, AB Sciex, USA) equipped with an electrospray ionization (ESI) source operating in positive mode and negative mode. The optimal conditions were set as follows: source temperature, 500°C; curtain gas (CUR), 30 psi; both ion sources GS1 and GS2, 50 psi; ion-spray voltage floating (ISVF), -4,000 V in negative mode and 5,000 V in positive mode; declustering potential, 80 V; and collision energy (CE), 20–60 V rolling for MS/MS. Data acquisition was performed with the data-dependent acquisition (DDA) mode. The detection was carried out over a mass range of 50–1,000 m/z.

### Multivariate Statistical Analysis

Multivariate statistical analysis was performed using ropes (Version 1.6.2, http://bioconductor.org/packages/release/bioc/html/ropls.html) R package from Bioconductor on Majorbio Cloud Platform (https://cloud.majorbio.com). Principal component analysis (PCA) using an unsupervised method was applied to obtain an overview of the metabolic data, and general clustering, trends, or outliers were visualized. All of the metabolite variables were scaled to unit variances before conducting the PCA. Orthogonal partial least squares discriminant analysis (OPLS-DA) was used for statistical analysis to determine global metabolic changes between comparable groups. All of the metabolite variables were scaled to Pareto scaling before conducting the OPLS-DA. Model validity was evaluated from model parameters R2 and Q2, which provide information for the interpretability and predictability of the model and avoid the risk of overfitting. Variable importance in the projection (VIP) was calculated in the OPLS-DA model. p values were estimated with paired Student’s t-test on single-dimensional statistical analysis.

### Statistical Analysis

The Wilcox rank-sum test was performed to analyze the statistical significance of the alpha diversity, KEGG (Kyoto Encyclopedia of Genes and Genomes database) module profiles, KO (KEGG ortholog), and different taxonomic levels (phylum, genera, and species) between the different cohorts. Linear discriminant analysis (LDA) effect size (LEfSe) analysis was used to identify the taxa or functional profiles most likely to explain differences between the biliary atresia and control groups. A linear discriminant analysis (LDA) score cutoff of 2.0 indicated a significant difference. The Spearman correlation test was conducted to investigate the relationship between the clinical parameters, microbial composition, microbial metabolites, and bile acid. We drew a heat map through the R software corrplot package/gplots package to exhibit the results. Differences were considered significant at *p* < 0.05 or false discovery rate (FDR) < 0.1.

## Results

### Characteristics of Gut Microbiota Profiles in Early-Stage Biliary Atresia

**Figure 1:**
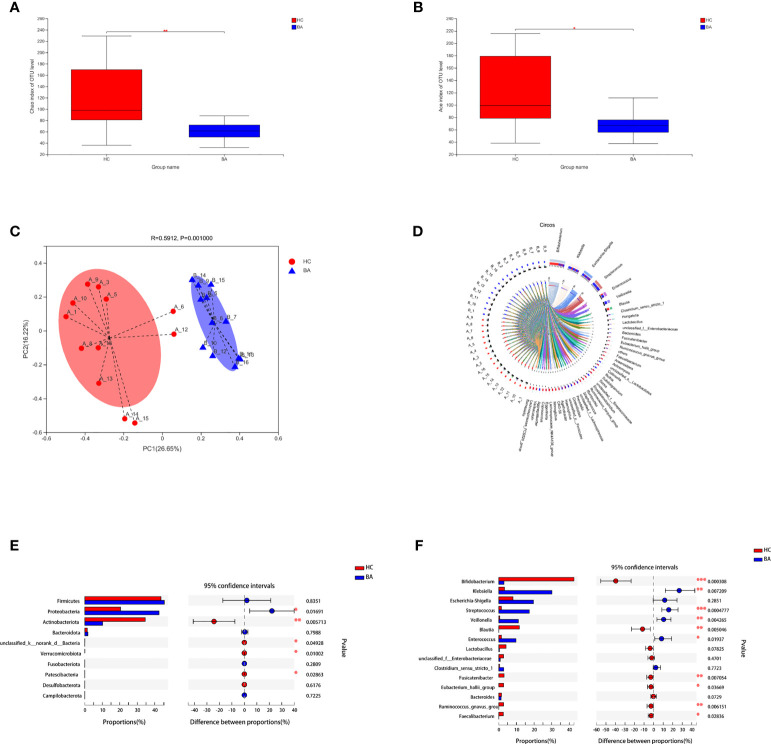
**(A, B)** Community richness (Chao and ace) of gut microbiota in patients with early-stage biliary atresia and controls (BA, biliary atresia; HC, controls) (*P < 0.05, **P < 0.01). **(C)** Beta diversity (principal coordinates analysis based on the Bray–Curtis distance of species abundance) between the two groups (BA, biliary atresia; HC, controls). **(D)** Correlation between fecal microbiota structure and samples. **(E, F)** Microbiota with significantly different abundances at the phylum and genus level were identified (BA, biliary atresia; HC, controls) (*P < 0.05, **P < 0.01, ***P < 0.001).

The indices of community richness (Chao and ace) were significantly decreased in patients with biliary atresia compared with controls (**Figures 1A, B**, *p* < 0.05). Principal coordinate analysis demonstrated differences in the microbiome structure between the two groups (**Figure 1C**, R = 0.2825, *p* = 0.001).

**Figure 1D** shows the correlation between fecal microbiota structure and samples. Proteobacteria were significantly increased in patients with biliary atresia, whereas Actinobacteria and Verrucomicrobia were decreased (**Figure 1E**). *Klebsiella*, *Streptococcus*, *Veillonella*, and *Enterococcus* were enriched in patients with biliary atresia, while *Bifidobacterium* and *Blautia* were enriched in controls (**Figure 1F** and **Table S1**).

### Characteristics of Gut Microbiota Profiles in Later-Stage Biliary Atresia

We investigated the structural features of gut microbiota in patients with end-stage liver disease and biliary atresia. The overall fecal microbiota community structure was significantly different between the later-stage biliary atresia and matched control groups (Anosim, R:0.317, *p* = 5 × 10^−4^; Adonis, R: 0.106, *p* = 0.003; principal coordinate analysis, *p* = 0.007).

**Figure 2:**
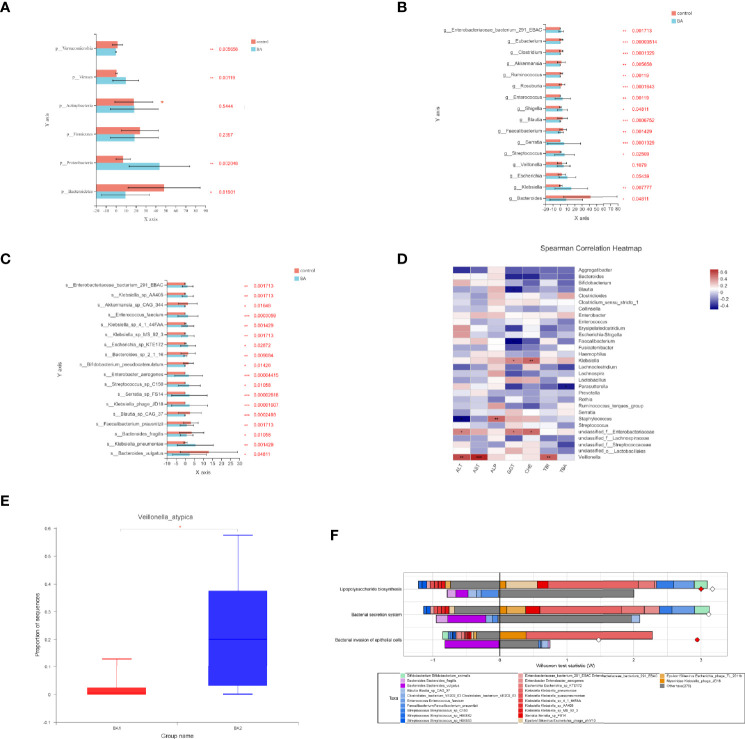
**(A–C)** Microbiota enriched in the different groups (patients with later-stage biliary atresia and controls) at the phylum, genus, and species levels (*p < 0.05, **p < 0.01, ***p < 0.001). Different colors represent different groups. Orange denotes the control group and blue biliary atresia (BA). **(D)** Correlations between liver function indicators and gut microbiome in different stages of biliary atresia (Spearman correlation test). The x-axis represents the environmental factors, and the y axis the species. The depth of the color indicates the correlation between species and environmental factors. Blue denotes negative correlation and red positive correlation (*p < 0.05, **p < 0.01, ***p < 0.001). **(E)** Changes in the abundance of *V. atypica* in early and later biliary atresia (BA1, early-stage biliary atresia; BA2, later stage biliary atresia). **(F)** FishTaco analysis of species contribution to metabolic pathways. The x-axis represents the Wilcoxon test statistic scores, and the y-axis the related functions. The driving factors for each differential function transformation were divided into four parts, represented by a histogram in two directions. Gut microbiota in the biliary atresia group drove the increase in the corresponding functional abundance (top right). Gut microbiota in the biliary atresia group inhibited the proliferation in the related practical quantity (top left). Gut microbiota in the control group drove the increase in the related functional mass (bottom right). Gut microbiota in the control group inhibited proliferation in the corresponding available abundance (bottom left). Different color bars represent the related species. The longer the bar, the greater the driving or inhibitory effect of the species on the corresponding function.

Proteobacteria were significantly increased while Bacteroidetes and Verrucomicrobia were decreased (**Figure 2A**). In addition, species enriched in the different groups at the genus and species levels are displayed in **Figures 2B, C**. *Klebsiella*, *Streptococcus*, *Veillonella*, and *Enterococcus* were the dominant species in patients with biliary atresia (**Table S2**). Additionally, we initially found several elevated viruses and fungi in biliary atresia individuals (**Table S2**). We concluded that *Klebsiella*, *Streptococcus*, *Veillonella*, and *Enterococcus* were dominant in the progression of biliary atresia.

### Relationship Between Gut Microbiota and Liver Function Indicators

To identify correlations between liver function indicators and gut microbiome in different stages of biliary atresia, we performed the Spearman correlation test (**Table S3**). The top 30 most abundant microorganisms were selected to analyze with liver indicators (**Figure 2D**). *Klebsiella*, *Veillonella*, *Staphylococcus*, and unclassified Enterobacteriaceae were positively correlated with liver enzymes (*p* < 0.05). Especially, *Veillonella* had a highly positive correlation with liver enzymes and bilirubin (*p* < 0.01). Furthermore, we discovered that *Veillonella atypica* was more highly elevated in the later than in the early stage of biliary atresia (22.71% *versus* 2.115%, **Figure 2E**). The above data indicated a strong correlation between *Klebsiella*, *Veillonella*, Enterobacteriaceae, and the progression of biliary atresia, especially *V. atypica*.

### Characteristics of Gut Microbiome’s Functional Profiles in Biliary Atresia

**Figure 3:**
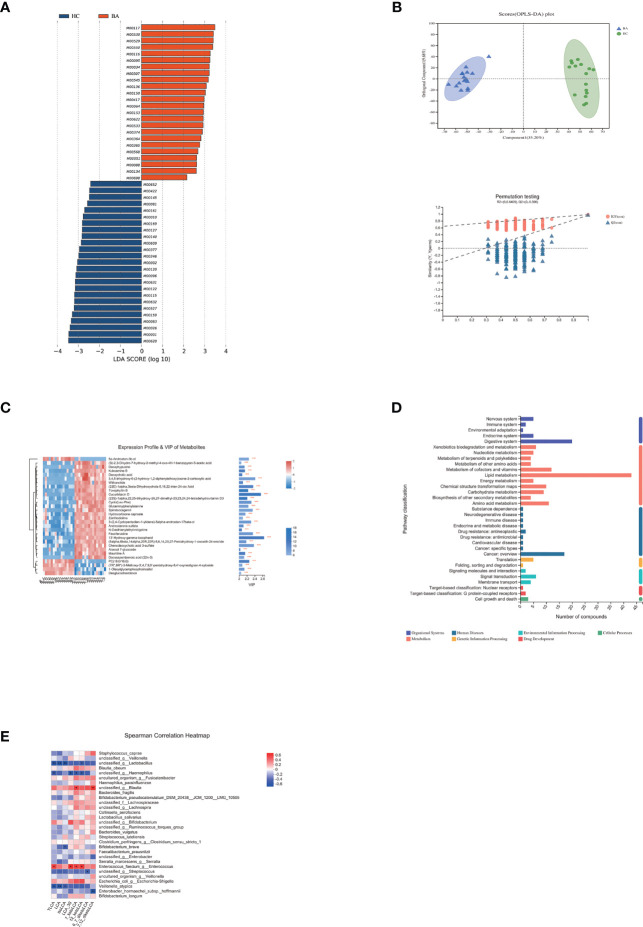
**(A)** LEfSe analysis of gut microbiota functional profiles between the control and biliary atresia groups (BA, biliary atresia; HC, controls). Histogram of the LDA scores calculated for a differential abundance of functional profiles in the two groups. LDA score cutoff of 2.0 indicated a significant difference. Different colors represent different groups. **(B)** Significant differences in metabolite composition between the biliary atresia and control groups identified by OPLS-DA (above: OPLS-DA map; below: model verification map of OPLS-DA; BA: biliary atresia; HC: controls). OPLS-DA map: the first prediction of Comp1 is mainly the decomposition degree, and the first orthogonality of orthogonal Comp1 is the decomposition degree. Model verification map of OPLS-DA: the x-axis represents the replacement retention of the replacement test; the y-axis represents the R2 (red dot) and Q2 (blue triangle) replacement test values. The two dashes represent the regression lines of R2 and Q2, respectively. **(C)** Heatmap of differential metabolites between the two groups (VIP > 2, *p* < 0.05). The color represents the relative abundance of the metabolites in the samples. On the right is the VIP bar graph of metabolites. The length of the bar represents the contribution value of the metabolite to the difference between the groups (*p < 0.05, **p < 0.01, ***P < 0.001). **(D)** KEGG pathways on level 1 and level 2 are related to differential metabolites. The ordinate is the name of pathway level 2, and the abscissa is the number of metabolites related to the pathway. Different colors represent different pathways on level 1. **(E)** Associations of differential lithocholic acid and derivatives with top 30 genera in abundance. The depth of the color indicates the correlation between species and environmental factors. Blue denotes negative correlation and red positive correlation (*p < 0.05, **p < 0.01, ***p < 0.001).

We annotated the catalogs using the KEGG database to investigate the gut microbiome’s functional profiles (https://www.kegg.jp/). Pathogen-associated molecular patterns, including capsular polysaccharide transport system (K10107 and K09688), LPS biosynthesis (K05790), adhesins (K13735), adhesin transport system (K12543 and K12542), and bacterial secretion system were all elevated in the biliary atresia group (**Table 1**). Combined enriched species and function analysis showed that *Klebsiella* spp. and Enterobacteriaceae played a more prominent role in pathways involving the bacterial invasion of epithelial cells, LPS biosynthesis, and bacterial secretion (**Figure 2F**). Besides, ketone body biosynthesis (M00088), polyamine biosynthesis (M00134), GABA biosynthesis (M00136), aromatic amino acid metabolism (M00533, M00545), and branched-chain amino acid metabolism (isoleucine biosynthesis, M00570) were enriched in the biliary atresia group (**Figure 3A**, **Table S4**).

**Table 1:** Elevated or decreased gut microbiome’s functional profiles in biliary atresia individuals.

KO ID	Description	Median (control)	Median (biliary atresia)	*p* value
K10107	Capsular polysaccharide transport system permease protein	0	3.27 × 10^-6	0.0002
K09688	Capsular polysaccharide transport system permease protein	0	9.17 × 10^-7	0.002
K05790	Lipopolysaccharide biosynthesis protein WzzE	3.63 × 10^-6	1.18 × 10^-4	0.001
K13735	Adhesin/invasin	6.99 × 10^-6	5.94 × 10^-5	0.01
K12543	Outer membrane protein, adhesin transport system	5.98 × 10^-7	8.92 × 10^-6	0.01
K12542	Membrane fusion protein, adhesin transport system	6.03 × 10^-7	1.43 × 10^-5	0.03
K00290	Saccharopine dehydrogenase	1.78 × 10^-4	5.75 × 10^-6	0.0002
K00640	Serine O-acetyltransferase	4.18 × 10^-4	3.04 × 10^-4	0.02
K09470	Gamma-glutamylputrescine synthase	5.51 × 10^-7	4.84 × 10^-5	0.0009
K09471	Gamma-glutamylputrescine oxidase	4.31 × 10^-6	9.08 × 10^-5	0.001
K09472	Gamma-glutamyl-gamma-aminobutyraldehyde dehydrogenase	1.00 × 10^-6	6.70 × 10^-5	0.0003
K09473	Gamma-glutamyl-gamma-aminobutyrate hydrolase	7.19 × 10^-7	5.83 × 10^-5	0.004
K01442	Choloylglycine hydrolase	3.03 × 10^-4	8.27 × 10^-5	0.0005

### Differential Metabolites Between Patients With Biliary Atresia and Controls

We performed non-targeted metabolomic profiling of stools from patients with biliary atresia and controls. Orthogonal partial least squares discriminant analysis (OPLS-DA) showed pronounced metabolic alterations between the two groups [**Figure 3B**, R^2^Y = (0, 0.6409), Q^2^ = (0, −0.396)]. A total of 19,817 differential peaks were selected, including 10,635 peaks in positive mode and 9,182 peaks in negative mode (**Table S5**). OPLS-DA identified 289 and 347 metabolites enriched in the biliary atresia and control groups, respectively. Differential metabolites among the top 30 are shown in **Figure 3C** (VIP > 2, *p* < 0.05) (**Table S6**).

Differential metabolites among the two groups were summarized and mapped into their biochemical pathways through metabolic enrichment and pathway analysis based on database search (KEGG, http://www.genome.jp/kegg/). Here, altered metabolites were mainly involved in lipid metabolism, digestive system, metabolism of cofactors and vitamins, and amino acid metabolism (**Figure 3D**). Bile acids are the primary metabolites involved in lipid metabolism and the digestive system. We detected a decrease in 15 bile acids and an increase in 2 bile acids in biliary atresia individuals (**Table S7**). Lithocholic acid (LCA) and its derivatives have been reported toxic to the liver (28). However, its abundance was similar between the two groups. The Spearman correlation test showed that *Enterococcus faecium* had an enormously positive relationship with taurolithocholate (TLCA), 12-ketolithocholic acid (12-ketoLCA), 7-ketolithocholic acid (7-ketoLCA), and lithocholic acid 3 sulfate (LCA-3S) (**Figure 3E**). Regarding differential metabolites involved in the metabolism of cofactors and vitamins, we listed changed vitamin A and D products in **Table S3**.

**Figure 4:**
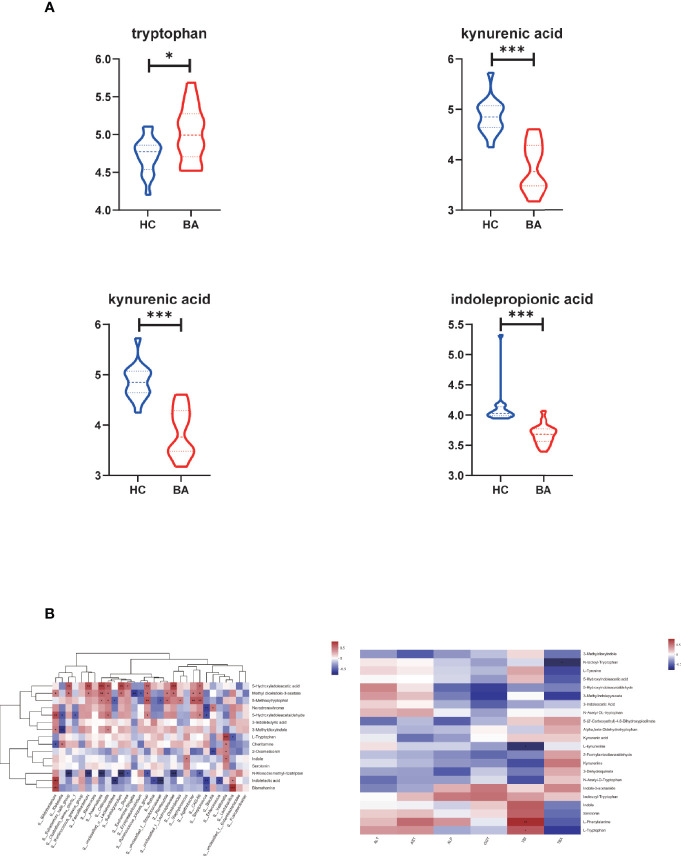
**(A)** Differential metabolites related to tryptophan metabolism in biliary atresia and control groups (*P < 0.05, ***P < 0.001) (BA, biliary atresia; HC, controls). **(B)** The relationship between altered species, metabolites, and liver function indicators. Left: Associations of differential metabolites with top 30 genera in abundance. Right: Associations of differential metabolites with liver function indicators (*P < 0.05, **P < 0.01, ***P < 0.001).

In amino acid metabolism, there was a significant change in metabolites involved in aromatic amino acid metabolism (especially tryptophan metabolism). We found that the abundance of tryptophan accumulated in biliary atresia individuals while kynurenic acid, kynurenine, and indole propionic acid were consumed (**Figure 4A**). The Spearman correlation test was performed to investigate the relationship between altered species, metabolites, and liver function indicators (**Figure 4B**). It displayed that *Veillonella* was strongly positive with tryptophan abundance. *Lactobacillus* was negative and positive with tryptophan and indoleacetic acid. Further, tryptophan and kynurenine were positively and negatively associated with total bilirubin concentration (TBIL). Based on these results, we speculated that there might be a relationship between *Veillonella*, tryptophan metabolism, and liver injury in biliary atresia individuals.

## Discussion

Various studies have been demonstrated that gut microbes are involved in the occurrence and development of many diseases. The previous study has suggested that the progression of dysbiosis can be associated with worsening liver disease (29). Experiments in mice also show that intestinal microbes can aggravate bile acid damage to liver cells (30). Besides, microbiota-derived metabolites, notably bile acids, short-chain fatty acids, tryptophan, and indole derivatives, have been strongly implicated in the pathogenesis of metabolic disorders (31).

Biliary atresia is the most common disease of cholestatic liver disease in children. It has been observed that the intestinal microbiome is maladjusted in it (24, 26). However, critical questions on microbiome-linked disease states and their relationship with disease course remain unexplored. Moreover, the attributes of metabolites and their relationship with intestinal microbiota and liver damage have also not been investigated. Based on this, we aimed to (1) describe the gut microbial composition in the early and later stages of biliary atresia; (2) search for microorganisms that changed significantly during disease progression and explored the potential relationship between liver function index and targeted bacteria; and (3) analyze the differential microbial function and fecal metabolites in biliary atresia and explore their relationship with gut microbiome and liver damage.

Overall, patients with biliary atresia showed a lower level of microbial community richness compared to controls. Besides, the microbial structure was visibly separated between the two groups, whether in the early or later stage, which indicated that gut microbiome disorder occurred in biliary atresia. Depleted bile acids in the gut and fibrosis in the liver were the main factors contributing to this outcome. Concerning species composition, Proteobacteria abundance increased significantly in the early and late stages of biliary atresia. Proteobacteria contain various known human pathogens. Their abnormal reproduction is often associated with an increase in epithelial oxygen availability. It is therefore considered a marker of inflammation and epithelial dysfunction (32). Subsequently, the biliary atresia-enriched Enterobacteriaceae family, which comprises *Klebsiella*, *Shigella*, *Salmonella*, and *Escherichia*, is an ordinary member of the Proteobacteria pathogenic bacteria in biliary tract infection (33). Adhesins (K13735, enriched in biliary atresia), as biological macromolecules on the surface of these bacteria, played an essential role in bacterial colonization through adhesion to biliary tract cells. By analyzing the gut microbial composition in different disease stages, we found that *Klebsiella*, *Streptococcus*, *Veillonella*, and *Enterococcus* were always dominant and may serve as potential biomarkers. On this basis, combined functional metabolism and clinical indicators showed that *Klebsiella* spp. and *V. atypica* played a significant role in the evolution of biliary atresia. It has been reported that *K. pneumoniae* can disrupt the epithelial barrier and initiate bacterial translocation and hepatitis through the virulence factors, capsular polysaccharide transport, and bacterial secretion systems (34, 35).

Subsequently, a series of reactions such as cytokine release and inflammation would have occurred. It is consistent with higher IL-2, IL-4, IL-6, IL-10, TNFα, and IFN-γ in patients with biliary atresia (**Table 2**). A high level of *K. pneumoniae* may be a stimulating factor for gastrointestinal perforation after transplantation, although the cause of this remains unclear. The risk factors include previous laparotomy, long duration of surgery, subsequent laparotomy, portal vein thromboembolism in the early period, treatment with high-dose steroids, and CMV infection (36). Excluding surgery-related factors, some children with biliary atresia developed bowel perforation after transplantation in our center.

**Table 2:** Results of cytokines between the control and biliary atresia groups.

Inflammatory cytokines	Biliary atresia (min, max)	Control (min, max)	*p* value
IL-2, pg/mL	73.63 (0.96, 262.02)	3.58 (0.96, 5.32)	<0.05
IL-4, pg/mL	39.47 (3.6, 143.85)	2.22 (1.77, 11.09)	<0.05
IL-6, pg/mL	70.87 (7.38, 233.53)	2.98 (1.23, 14.92)	<0.05
IL-10, pg/mL	25.66 (2.56, 61.84)	0.86 (0.27, 4.28)	<0.05
TNFα, pg/mL	31.55 (2.2, 117.86)	2.09 (1.11, 3.26)	<0.05
IFN-r, pg/mL	85.38 (2.1, 341.05)	2.1 (2.09, 2.11)	<0.05

IL-2, interleukin-2; IL-4, interleukin-4; IL-6, interleukin-6; IL-10, interleukin-10; TNFα, tumor necrosis factor α; IFN-γ, interferon-γ.

*Enterococcus* was another dominant genus in biliary atresia individuals, whether in the early or later stage. It comprises a ubiquitous group of Gram-positive bacteria that are of great relevance to healthcare-associated infections (37). Among them, *E. faecium* was one of the most abundant enterococcal species. In primary sclerosing cholangitis (PSC), it has been demonstrated that TLCA levels strongly correlated with *Enterococcus* abundance (32). TLCA is one of the derivatives of lithocholic acid. Lithocholic acid and its conjugates are considered the most harmful bile acids (32, 38). They could cause segmental bile duct obstruction, destructive cholangitis, and periductal fibrosis (32). In the current study, most bile acids were reduced in biliary atresia individuals. However, the level of lithocholic acid and its derivatives was similar between controls and biliary atresia individuals. Significantly, we found a strong positive relationship between *E. faecium* and lithocholic acid derivatives. Based on the above, we believed that *E. faecium* promoted the progression of liver injury in biliary atresia through bile acid metabolism.

In the disease progression of biliary atresia, there was a significant increase in the abundance of *V. atypica*. *Veillonella* is a lactate-fermenting bacteria that generally reside in the oral cavity (39). Previous literature found that *Veillonella* abundance is related to the disease states of nonalcoholic steatohepatitis (NASH) (40). Moreover, Wei et al. also concluded that expansion of another species of *Veillonella* (*V. dispar*) was associated with the disease status of autoimmune hepatitis (AIH) (41). The present study discovered that the *V. atypica* concentration was strongly related to the liver enzyme in biliary atresia. We observed that *Veillonella* and tryptophan derivatives were associated with liver damage combined with gut microbiome and metabolites. It has been known that tryptophan dysmetabolism is associated with liver inflammation, steatosis, and insulin resistance (31). The kynurenic acid, kynurenine, and indole propionic acid products of tryptophan from different catabolism pathways (42). Among them, metabolites generated from kynurenine may regulate diverse cellular functions, including viability, adhesive and migratory properties, and inflammatory potential (43). Besides, defective catabolism for indole and its derivatives also contributes to intestinal permeability and LPS translocation (31). Consistently, the levels of tryptophan, kynurenic acid, kynurenine, and indole propionic acid were all significantly changed in this subject. Based on these, we believed that *V. atypica* was associated with the disease status in biliary atresia. Besides, tryptophan dysmetabolism also participated in the progress of the biliary atresia.

In addition to the growth of pathogenic bacteria, Bifidobacterium and some butyrate-producing bacteria like Faecalibacterium prausnitzii, Roseburia spp. (Roseburia faecis, Roseburia inulinivorans, Roseburia intestinalis, and Roseburia hominis), Eubacterium hallii, and Anaerostipes caccae were reduced in patients with biliary atresia (**Table S8**). Bifidobacterium and most butyrate-producing bacteria belong to the phyla Actinobacteria and Firmicutes, respectively (44). It could explain the decrease in Actinobacteria in patients with biliary atresia. Butyrate is a short-chain fatty acid that can reduce gut mucosal inflammation and protect the gut epithelial barrier integrity in several diseases (27, 45–48). These anti-inflammatory effects are partly associated with secreted metabolites capable of blocking nuclear factor-κB activation (49).

In conclusion, gut microbiota disorder occurred in patients with biliary atresia, whether in the early or later stage, and *Klebsiella*, *Streptococcus*, *Veillonella*, and *Enterococcus* were dominant. In addition, the liver damage of biliary atresia was directly or indirectly exacerbated by the interaction of enriched *Klebsiella* (*K. pneumoniae*), *Veillonella* (*V. atypica*), and *Enterococcus* (*E. faecium*) with dysmetabolism of tryptophan and bile acid.

## Data Availability Statement

The datasets presented in this study can be found in online repositories. The names of the repository/repositories and accession number(s) can be found below: NCBI SRA BioProject, accession no: PRJNA730640.

## Ethics Statement

The studies involving human participants were reviewed and approved by the Beijing Friendship Hospital, Capital Medical University (Approval ID: 2019-P2–131-02). Written informed consent to participate in this study was provided by the participants’ legal guardian/next of kin.

## Author Contributions

WS: study design, data collection, analysis, interpretation of the data, and manuscript writing. L-YS: study design, study supervision, and critical revision of the manuscript for important intellectual content. Z-JZ, LW, WQ, Z-GZ, YL, and H-MZ: clinical treatment assistance. WG: data collection. All authors contributed to the article and approved the submitted version.

## Funding

This study was supported by the National Natural Science Foundation of China (Grant No. 81570586).

## Conflict of Interest

The authors declare that the research was conducted in the absence of any commercial or financial relationships that could be construed as a potential conflict of interest.

## Publisher’s Note

All claims expressed in this article are solely those of the authors and do not necessarily represent those of their affiliated organizations, or those of the publisher, the editors and the reviewers. Any product that may be evaluated in this article, or claim that may be made by its manufacturer, is not guaranteed or endorsed by the publisher.

## Abbreviations

BA, biliary atresia; LCA, lithocholic acid; TLCA, taurolithocholate; 12-ketoLCA, 12-ketolithocholic acid; 7-ketoLCA, 7-ketolithocholic acid; LCA-3S, lithocholic acid 3 sulfate; CMV, cytomegalovirus; EBV, Epstein–Barr virus; KO, KEGG ortholog; KEGG, Kyoto Encyclopedia of Genes and Genomes; LEfSe, LDA effect size; LDA, linear discriminant analysis; LPS, lipopolysaccharide; OPLS-DA, orthogonal partial least squares discrimination analysis; VIP, variable importance in the projection; ALT, alanine aminotransferase; AST, aspartate aminotransferase; ALP, alkaline phosphatase; GGT, γ-glutamyl transferase; TBA, total bile acids; TBIL, total bilirubin; IL, interleukin; TNFα, tumor necrosis factor α; IFN-γ, interferon-γ; PELD, pediatric end-stage liver disease.
