# Supplementary material for: Association of Gut Microbiota and Metabolites With Disease Progression in Children With Biliary Atresia
Source: Front Immunol. 2021 Sep 23;12:698900. doi: 10.3389/fimmu.2021.698900 (PMC8495239; doi:10.3389/fimmu.2021.698900)
Supplement: Supplementary file 1 [file DataSheet_1.docx]

**Association of gut microbiota and metabolites with disease progression in children with biliary atresia**

Wei Song^1,3^, Li-Ying Sun^1,2,3*^, Zhi-Jun Zhu^1,3^, Lin Wei^1,3^, Wei Qu^1,3^, Zhi-Gui Zeng^1,3^, Ying Liu^1,3^, Hai-Ming Zhang^1,3^, Wei Guo^4^

**Author affiliations**

1.Liver Transplantation Center, National Clinical Research Center for Digestive Diseases, Beijing Friendship Hospital, Capital Medical University, 100000, Beijing, China

2.Department of Intensive Care Unit, Beijing Friendship Hospital, Capital Medical University, 100000, Beijing, China

3.Clinical Center for Pediatric Liver Transplantation, Capital Medical University, 100000, Beijing, China

4.Hangkong Hospital, China Capital University, Beijing, China *Correspondence:

Li-Ying Sun

***Correspondence to:**

Li-Ying Sun; 95 Yong’an Road, Xicheng District, Beijing, 100050, China; 010-8083-8160; [sunxlx@outlook.com](mailto:sunxlx@outlook.com).

Table S1 Clinical characteristics of study subjects

| Table S1 Demographic characteristics of the cohorts | | | | | | |
| --- | --- | --- | --- | --- | --- | --- |
| Characteristics | Early-stage biliary atresia and matched controls | | | Later stage biliary atresia and matched controls | | |
|  | Biliary atresia (n=16) | Control (n=16) | P value* | Biliary atresia (n=16) | Control (n=10) | P value* |
| Demographics | | | | | | |
| Age, months, median (min, max) | 1.8 (0.9, 2.9) | 1.5 (0.7, 2.6) | >0.05 | 7.8 (4.8, 21) | 8 (5, 20) | >0.05 |
| Gender, female (%) | 9 (56.25%) | 8 (50%) | >0.05 | 10 (62.5%) | 6 (60%) | >0.05 |
| BMI, kg/m2, median (min, max) | 17.21 (15.7, 19.8) | 18.39 (14.4, 20.28) | >0.05 | 16.94 (11.26, 21.33) | 18.79 (15.85, 20.09) | >0.05 |
| Hepatic function, median (min, max) | | | | | | |
| ALT, U/L | 165.35 (31.1, 588.1) |  |  | 151 (21, 439) | 14.85 (10.2, 20.1) | <0.05 |
| AST, U/L | 231.7 (80.8, 570.6) |  |  | 211.9 (45.4, 724.3) | 39.35 (27.2, 43.9) | <0.05 |
| ALP, U/L | 606.81 (245,193) |  |  | 610.5 (296, 1409) | 236.2 (192.6, 317) | <0.05 |
| GGT, U/L | 640.13 (44, 1532) |  |  | 180.5 (26, 980) | 11.3 (9, 16.3) | <0.05 |
| TBA, umol/L | 120.13 (74.3, 191.9) |  |  | 163.8 (57, 356) | 1.3 (0.3, 4.7) | <0.05 |
| TBIL, umol/L | 153.47 (110.1, 230.6) |  |  | 278.5 (9.02, 898.13) | 5.7 (2.5, 11.6) | <0.05 |

Abbreviations: ALT, alanine aminotransferase; AST, aspartate aminotransferase; ALP, alkaline phosphatase; GGT, γ-glutamyltransferase; TBA, total bile acids; TBIL, total bilirubin.

Table S2 Differential viruses and fungi between the control and biliary atresia groups

|  | Median (Control) | Median (BA) | *P* value |
| --- | --- | --- | --- |
| Viruses | | | |
| *Human mastadenovirus C* | 0 | 0.00000516661650337 | 0.04 |
| *Epsilon15likevirus* | 0.000008684298406155 | 0.000098422375772 | 0.04 |
| *Lambdalikevirus* | 0.000279224890329 | 0.002948512435955 | 0.04 |
| *Human herpesvirus 4* | 0 | 0.00329508078221548 | 0.03 |
| Fungi | | | |
| *Malassezia restricta* | 0 | 0.0000002145144299185 | 0.003 |
| *Candida albicans* | 0 | 0.000000015757121857 | 0.01 |
| *Candida auris* | 1.01×10^(-10) | 2.96×10^(-9) | 0.04 |
| *Candida parapsilosis* | 0 | 0.0000000231382380742 | 0.004 |
| *Aspergillus niger* | 5.6×10^(-9) | 1.88×10^(-8) | 0.01 |
| *Melampsora pinitorqua* | 0.0000000056374073433 | 0.0000002222827583745 | 0.01 |

Table S3 Changed abundance in Vitamin A and D products

|  | VIP (OPLS-DA) | FC (BA/HC) | *P* value |
| --- | --- | --- | --- |
| Vitamin A | | | |
| Retinol | 1.882280566 | 1.589930151 | 0.000000000032 |
| 9-cis-Retinoic acid | 1.594458987 | 1.882869693 | 0.00000000186 |
| Vitamin D | | | |
| Calcitroic acid | 1.620291442 | 1.604480353 | 0.0000000425 |
| 25-Hydroxyvitamin D3-26,23-lactone | 1.916269183 | 0.486138614 | 0.00000232 |
| 24,25-Dihydroxyvitamin D | 1.587102918 | 0.792810557 | 0.0000000000000141 |
| 1,25-Dihydroxyvitamin D3-26,23-lactone | 1.591783636 | 0.682315789 | 0.00000000381 |
